# Massive Left Ventricular Thrombus Causing Bilateral Posterior Cerebral Artery Stroke: A Case Report and Review of Literature

**DOI:** 10.7759/cureus.27585

**Published:** 2022-08-01

**Authors:** Ashali Jain, Asad Haider, Tyler S Jones

**Affiliations:** 1 Internal Medicine, University of Central Florida College of Medicine/HCA Florida North Florida Hospital, Gainesville, USA

**Keywords:** lvt, acute myocardial infarction, direct oral anticoagulation, post mi complications, posterior cerebral artery stroke, anti coagulation, left ventricular thrombus

## Abstract

Left ventricular thrombus (LVT) is a major complication of acute myocardial infarction (MI). Here, we describe the case of a 36-year-old female with a history of acute anterior MI six years prior to hospitalization, who presented with bilateral vision loss due to a bilateral embolic posterior cerebral artery (PCA) stroke in the setting of a 5.7 x 1.7 cm LVT. She underwent bilateral PCA thrombectomy, which led to improvement of her symptoms. Her LVT was managed non-surgically with apixaban and clopidogrel. Her case highlights the need for more medical education about LVT, as quick initiation of anticoagulation is essential in improving outcomes. We review the existing literature to explain the pathogenesis, diagnosis, and treatment of LVT.

## Introduction

Left ventricular thrombus (LVT) is a major complication of acute myocardial infarction (MI). Tissue necrosis from MI can lead to left ventricular (LV) dyskinesis, resulting in blood stasis within the LV cavity [1]. Although recent advances have led to improved survival in acute MI, postinfarction complications represent a significant cause of morbidity and mortality [2]. LVT remains common, with an estimated incidence of 15% in patients with acute MI [2]. Treatment has traditionally consisted of oral anticoagulation (OAC) plus dual antiplatelet therapy (DAPT), although recent evidence suggests single antiplatelet therapy plus OAC is associated with lower bleeding and similar efficacy as OAC plus DAPT [3-5]. LVTs must be monitored closely, as there is a potential for embolization that can increase morbidity and mortality. Here, we describe a case of a 36-year-old female, with a history of acute anterior MI six years prior to hospitalization, who presented with bilateral vision loss due to a bilateral embolic posterior cerebral artery (PCA) stroke in the setting of a 5.7 x 1.7 cm LVT.

## Case presentation

A 36-year-old female, with a past medical history significant for MI treated with angioplasty and percutaneous coronary intervention of the left anterior descending artery six years prior to presentation, presented with bilateral vision loss. She also complained of a severe headache but no other symptoms. Her other comorbidities include hypertension and type 2 diabetes mellitus. She reported being adherent to DAPT with aspirin and clopidogrel for one year after her stent implantation. Her family history was negative for early stroke or coronary artery disease (CAD). After her previous MI, she was recommended to follow up outpatient for hypercoagulable workup, but failed to do so. She denied using illicit substances or drinking alcohol, although she did smoke one half a pack per day of cigarettes since she was 15 years old. There was no prior history of miscarriages and she had not been taking hormone-containing contraceptives since her MI.

Her physical examination, with the exception of her loss of vision, was unremarkable and she was alert and oriented to person, place, and time. She followed commands and exhibited no motor deficits. Interestingly, even though she reported complete blindness, she was able to track physicians moving without being prompted.

On admission, complete blood count (CBC), complete metabolic panel (CMP), prothrombin time (PT)/international normalized ratio (INR), thyroid stimulating hormone (TSH), and lipid panel were all within normal limits. Urine toxicology screen was negative.

**Figure 1:**
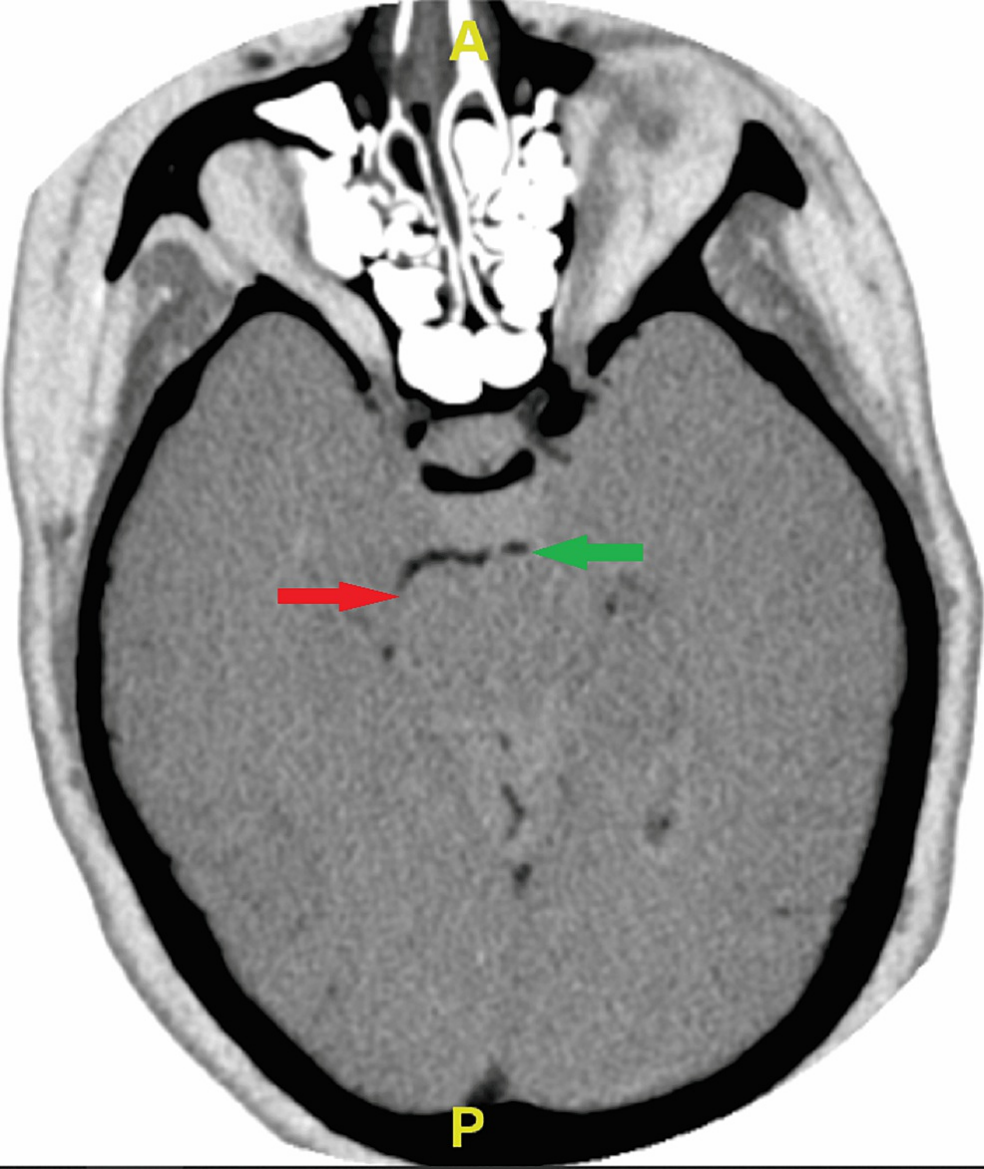
Inverted image of the CTA of the head and neck showing occlusion of the posterior cerebral arteries Axial view of the CTA of the head and neck. The areas of arterial occlusion are indicated as follows: red arrow indicates occlusion of the right posterior cerebral artery at the P3 level, while the green arrow indicates occlusion of the left posterior cerebral artery at the P2 level. Areas in black proximal to the occlusions indicate areas of normal blood flow. CTA: computed tomography angiography

**Figure 2:**
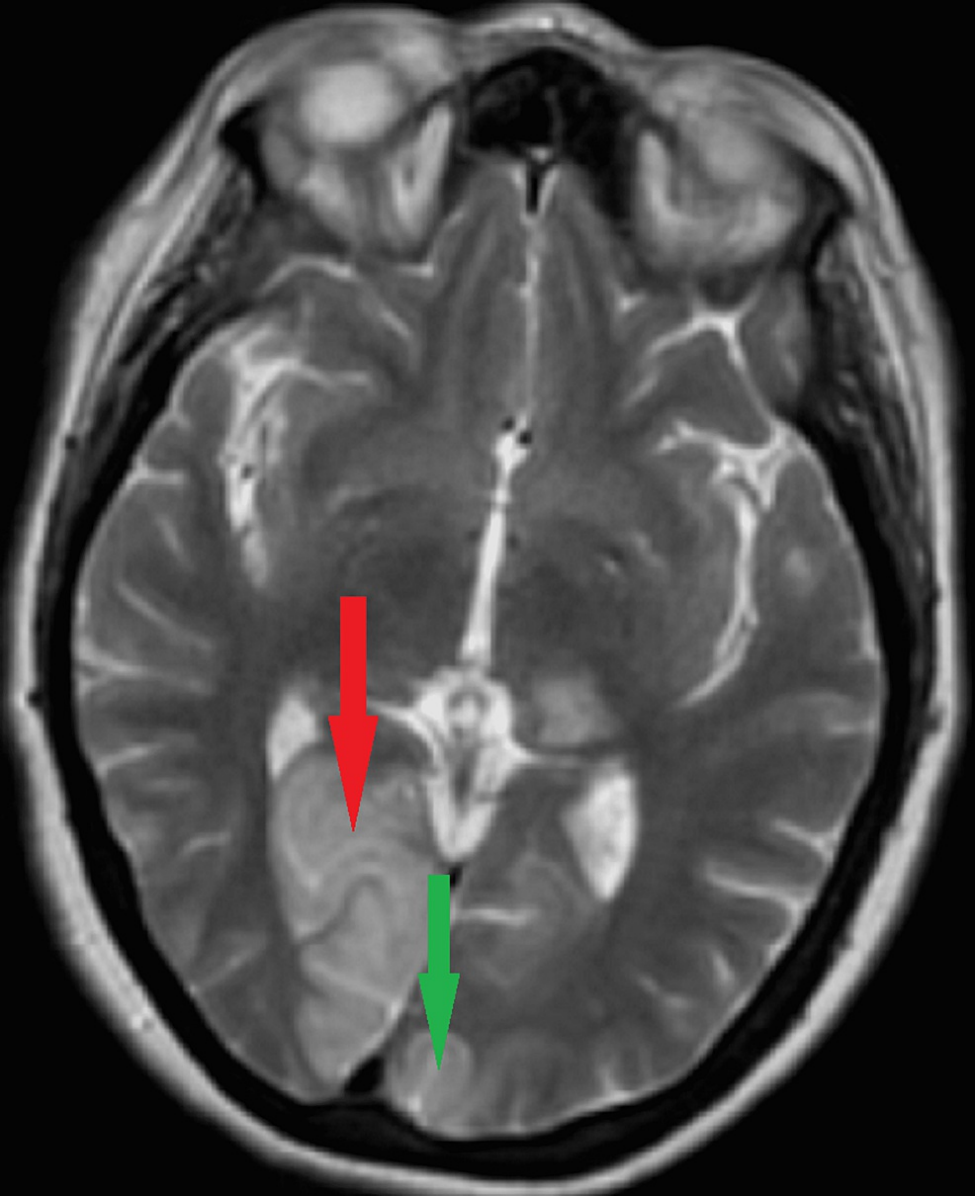
Representative T2 view of the MRI of the brain showing areas of infarction in bilateral PCA stroke Axial view of the MRI of the brain. The areas of infarction are indicated as follows: red arrow indicates right occipital lobe, while green arrow indicates left occipital lobe.

Computed tomography angiography (CTA) of the head and neck revealed occlusion of the left (one centimeter beyond the basilar artery) and right (P3 level) posterior cerebral arteries (Figure 1). Magnetic resonance imaging (MRI) demonstrated infarction within both occipital lobes, with no acute hemorrhage, midline shift, hydrocephalus, or herniation (Figure 2). She was not an alteplase candidate as her last known normal was over 4.5 hours prior to admission. The patient underwent neurointerventional biplane angiography and was found to have thrombi in both posterior cerebral arteries. She underwent successful mechanical thrombectomy. She was transported to the intensive care unit (ICU) for post-procedure observation, and follow-up imaging ruled out hemorrhagic conversion. She was started on intravenous heparin, atorvastatin, aspirin, and clopidogrel.

A transthoracic echocardiogram showed a 5.7 x 1.7 cm large left ventricular heterogeneous mobile thrombus attached to an immobile apex (Video 1). An ejection fraction of 60-65% was also noted. The remainder of the echocardiogram was largely unremarkable. Cardiothoracic surgery evaluated the patient and determined there was no indication of emergent surgery. After cardiology evaluated the patient, she was transitioned from the heparin drip to long-term therapy with clopidogrel and apixaban. After a few days of observation, she was discharged and scheduled for a follow-up with a cardiologist for monitoring of her LVT.

**Video 1:** Representative transthoracic echocardiogram showing thrombus in the left ventricle

## Discussion

Etiology and risk factors

LVT is a well-known complication of myocardial infarction and significantly increases the risk of mortality as well as complications from further embolization [6-8]. The pathophysiology has been well researched and the etiology of these masses arises from a variety of factors. In the setting of myocardial infarction, it is the cell necrosis and simultaneous hypokinesis that results in stasis of the blood and subsequent thrombus formation [8]. Similarly, in atrial fibrillation, the stasis of blood flow leads to an increased risk of hypercoagulability. Though rates vary, studies suggest almost a 19% increased risk of thromboembolism without proper anticoagulation therapy [9].

As vital as it is to understand the mechanism of thrombus formation, it is equally vital as clinicians to recognize patients at increased risk of these events. Diagnostic tools, such as transthoracic echocardiography (TTE), risk stratification tools, and cardiac computed tomography (CT), have been studied to determine the efficacy of predicting adverse events including thrombus formation. Currently, TTE is the initial tool of choice for detecting LVT. TTEs have demonstrated high sensitivity and specificity in terms of detection, and are reliably able to rule out thrombus formation [10]. Other imaging modalities have been explored to improve detection rates. For instance, contrast-induced imaging does significantly improve the characterization of the vascular composition of masses [11]. In addition, CT angiography (CTA) and MRI have demonstrated high specificity and sensitivity in detecting not only LVTs but also left atrial thrombi [12]. It is thought that cardiac MRI may additionally aid in detecting smaller mural thrombi or those not easily visualized on TTEs [13,14]. 

Oral anticoagulation therapy

LVTs have been found to persist even in the setting of percutaneous coronary intervention in the post-MI setting [15]. Current guidelines recommend the use of DOACs, as evidence is largely supportive of efficacy and resolution of thrombosis [16-19]. Vitamin K antagonists have traditionally been used, but due to the difficulties of monitoring the INR, it can be difficult in the setting of other drug and food interactions [20]. Anticoagulation is usually discontinued after three to six months of treatment and subsequent echocardiograms showing resolution of the thrombus [1].

DAPT is also another vital component of the management of newly-diagnosed LVTs. Currently, new evidence suggests that the efficacy of monotherapy antiplatelet therapy may be just as efficacious as DAPT, with decreased risk of bleeding [5]. The duration of such therapy is still variable and is currently largely dependent on clinician discretion.

## Conclusions

In conclusion, we present a case of a patient with massive LVT causing a bilateral embolic PCA stroke. We have also reviewed the existing literature to explain the pathogenesis, diagnosis, and treatment of LVT. LVT remains a significant complication of postinfarction morbidity and mortality, and it is important to improve awareness of this syndrome. As research and education about LVT increases, we anticipate seeing an improvement in patient outcomes.
